# Impacts of the COVID-19 pandemic on the lives of adolescents living with HIV aged 10–15 years and their families in Vietnam

**DOI:** 10.1186/s12889-026-27867-3

**Published:** 2026-05-26

**Authors:** Khanh Toan Tran, Quynh Trang Pham, An Van Pham, Thi Kim Chuc Nguyen, Huynh Linh Dinh, Tuan Quy Du, Chau Viet Do, Van Lam Nguyen, Thi Loc Nguyen, Kim Dung Khuong, Linus Olson, Mattias Larsson

**Affiliations:** 1 Department of Family Medicine, Hanoi Medical University, Hanoi, Vietnam; 2 FilaBavi HDSS, Hanoi Medical University, Hanoi, Vietnam; 3 Training and Research Academic Collaboration (TRAC) – Sweden – Vietnam, Hanoi, Vietnam; 4 Cardiology Department, Hanoi Medical University, Hanoi, Vietnam; 5 Children Hospital 1, Ho Chi Minh City, Vietnam; 6 Children Hospital 2, Ho Chi Minh City, Vietnam; 7 Tropical Diseases Center, Vietnam National Children’s Hospital, Hanoi, Vietnam; 8 Hai Phong Center for Disease Control, Hai Phong, Vietnam; 9 Quang Ninh General Hospital, Quang Ninh, Vietnam; 10 Department of Public Health Sciences, Karolinska Institutet, Stockholm, Sweden; 11 Department of Women’s and Children’s Health, Karolinska Institutet, Tomtebodavägen 18A, 8fl, Stockholm, 17176 Sweden

**Keywords:** Adolescents living with HIV, COVID-19 pandemic, Perceived impact, Antiretroviral therapy (ART), Multiple life domains, Vietnam

## Abstract

**Background:**

Adolescents living with HIV are particularly vulnerable to disruptions caused by public health emergencies. This study assessed the perceived impacts of the COVID-19 pandemic and associated factors on adolescents living with HIV aged 10–15 years and their families in Vietnam.

**Methods:**

A cross-sectional study was conducted among 587 adolescents living with HIV 10–15 years who were receiving antiretroviral therapy (ART) at five central and provincial hospitals between June 2020 and December 2021 using a structured questionnaire. Perceived impacts of COVID-19 across multiple life domains were measured using a five-point Likert scale. To identify associated factors chi-square tests and multivariate logistic regression analyses were performed.

**Results:**

Overall, 35.9% of participants reported a significant overall perceived impact of the COVID-19 pandemic. Among adolescents residing in Ho Chi Minh City the odds of experiencing a significant overall perceived impact were higher compared to (aOR 18.8, 95% CI 5.9–60.0), Quang Ninh (aOR 5.9, 95% CI 1.2–28.6) and other provinces outside the hospitals’ catchment areas (aOR 4.9, 95% CI 1.6–14.9). Increased odds were also found among those taking ART twice daily (aOR 1.9, 95% CI 1.1–3.5); those living in households with 3–4 members (aOR 2.9, 95% CI 1.3–6.5) or ≥ 5 members (aOR 2.7, 95% CI 1.2–6.1); those without home ownership (aOR 2.0, 95% CI 1.1–3.3); and those whose caregivers reported a need for social support (aOR 4.6, 95% CI 2.8–7.7).

**Conclusions:**

Substantial impacts of the COVID-19 pandemic were reported among adolescents living with HIV and their families, underscoring the vulnerability of this population during public health emergencies. Interventions addressing both individual- and family-level needs are needed to strengthen resilience and mitigate the effects of future pandemics.

**Supplementary Information:**

The online version contains supplementary material available at 10.1186/s12889-026-27867-3.

## Introduction

The COVID-19 pandemic, caused by the SARS-CoV-2 coronavirus, has had far-reaching consequences for various aspects of society, including mental health and socio-economic conditions [1, 2]. Patients with chronic illnesses in general, and particularly individuals living with HIV and receiving antiretroviral therapy (ART), are among groups most severely affected by the pandemic [3].

In addition to the challenges posed by isolation and lockdown measures aimed at limiting viral transmission, people living with HIV have faced increased stigma, community fear, and barriers to accessing ART services [4, 5]. Among this population, adolescents are of particular concern due to their psychophysiological vulnerability [6]. The combined challenges of living with HIV and navigating pandemic-related disruptions may place adolescents at heightened risk of adverse social, psychological, and economic impacts. It is crucial to understand the specific effects of the pandemic on this vulnerable group in order to develop targeted interventions and support systems.

In Vietnam, the first confirmed case of COVID-19 was reported in January 2020. During the initial phase of the pandemic, Vietnam maintained low levels of community transmission through early border closures, rigorous contact tracing, and centralized quarantine across the first three waves. This situation changed significantly during the fourth wave (April–September 2021), when widespread transmission driven by the Delta variant accounted for nearly all confirmed infections nationwide (99.7%), with Ho Chi Minh City (HCMC) and surrounding southern provinces emerging as the epicentre and bearing a disproportionate burden of cases under strict lockdown measures [7]. As of 2 January, 2022, Vietnam had reported 1,760,188 locally acquired COVID-19 cases across all 63 provinces, with HCMC alone accounting for 504,197 cases (28.6%) [8]. In contrast, many other localities, particularly smaller provinces and those in northern Vietnam, experienced more limited transmission and lower case counts, highlighting substantial regional heterogeneity in the timing and intensity of COVID-19 spread [7, 8].

Despite substantial regional heterogeneity in the COVID-19 epidemic and the high burden observed in Ho Chi Minh City, evidence remains scarce on COVID-19 exposure and its broader, multidimensional impacts among adolescents living with HIV in Vietnam. To date, little is known about how the pandemic has affected their daily lives, antiretroviral treatment experiences, and family well-being in paediatric care settings. Addressing this gap is essential for informing interventions that strengthen resilience and ensure continuity of HIV care during future epidemics, pandemics and public health crises.

As part of the HIVCHI-2 project (“Interventional study on the impacts of peer supporters on enhancing the effectiveness of ART in adolescents living with HIV”), we conducted this study to assess the perceived impacts of the COVID-19 pandemic on adolescents living with HIV aged 10–15 years and their families in Vietnam, and to identify factors associated with experiencing a significant overall perceived impact. This was based on participants’ self-rated overall assessment of how strongly the pandemic had affected their lives and family circumstances across all assessed aspects collected.

## Methods

### Study design and setting

This cross-sectional study was nested within the HIVCHI-2 longitudinal cohort (Proposed protocol in Appendix 1, (There where more sites added and we could not reach all endpoints due to covid and change of policies in Vietnam)). During the study period in Vietnam, most children and adolescents living with HIV were concentrated in major paediatric hospitals, as ART services at primary-level facilities were limited and patient numbers were small and geographically dispersed. Accordingly, the study was conducted at five central and provincial paediatric hospitals with large numbers of adolescents receiving ART, including Vietnam National Children’s Hospital (Hanoi), Hai Phong Children’s Hospital, Quang Ninh General Hospital, Children’s Hospital 1, and Children’s Hospital 2 (Ho Chi Minh City).

### Participants

Within the HIVCHI-2 project, adolescents living with HIV aged 10–15 years who had been receiving ART for at least six months at one of the five participating paediatric hospitals were enrolled together with their primary caregivers between June and October 2020 and followed for 12 months. This age group was selected because adolescents aged 10–15 years are primarily managed in paediatric hospitals, whereas those aged 16 years and older are typically transitioned to decentralized care at district-level health facilities. Participants from this cohort constituted the analytic sample for the present study, with COVID-19 impacts being assessed at the end of the project after a 12-month follow-up. Adolescents who were temporarily referred from other ART facilities, transferred to other facilities before completing follow-up, or declined to participate were excluded. This age range represents a developmental stage characterized by greater caregiver dependence and vulnerability to disruptions in healthcare, education, and household socioeconomic conditions, allowing a more homogeneous assessment of COVID-19 impacts.

### Sampling and sample size

The minimum required sample size was calculated using the standard formula for estimating a proportion in a cross-sectional study:

$$n=\frac{Z^2_{1-{\alpha}/{2}}*\mathrm{p}\left(1-\mathrm{p}\right)}{\mathrm{d}^2}$$

where: *n* is the minimum sample size; $\:{Z}_{1-\alpha/2}^{2}\:$is the critical value of the normal distribution at a two-sided significance level of 0.05 ($\:{Z}_{1-\alpha/2}^{2}=1.96$), *p* is the expected proportion of adolescents experiencing a significant COVID-19 impact, and *d* is the margin of error for *p*.

Because no previous data were available on COVID-19 impacts among adolescents living with HIV in Vietnam, the expected prevalence was conservatively assumed to be 30% (*p* = 0.30) using the most relevant published evidence available at the time, based on findings reported by Ranjitkar et al. from a study of pandemic-related family impacts in Nepal [9]. Using a 5% margin of error and a 95% confidence level, the required minimum sample size was estimated at 323 participants. It should be noted that this assumption was based on a non-comparable population; however, the estimate was used as a pragmatic planning assumption to avoid underestimating the required sample size for this exploratory study. All eligible adolescents were invited to participate, and the final analytical sample (*n* = 587) substantially exceeded this minimum, supporting adequate precision and robustness of our analyses.

### Data collection

Baseline demographic, socioeconomic, and clinical information was collected at enrolment and updated quarterly throughout the HIVCHI-2 project using face-to-face interviews and medical record reviews. Perceived impacts of COVID-19 over the preceding 12 months were subsequently assessed at the end of the project using a structured questionnaire. The questionnaire was adapted from instruments used in previous studies conducted among vulnerable populations and individuals living with HIV and other chronic conditions [5, 9–12]. It was contextualized for Vietnam and pilot-tested among 10 participants prior to implementation.

Data collection was initially scheduled for completion by October 2021 but was extended until December 2021 due to disruptions related to the COVID-19 pandemic, including delays in follow-up visits and interview scheduling. Trained nurses working at ART outpatient units collected the data, which were entered directly into electronic data capture devices under supervisory oversight. Quality control procedures were implemented to ensure data completeness and accuracy.

### Variables and measurements

#### Dependent variables

COVID-19 impacts comprised perceived impacts across multiple life domains and direct exposure outcomes, which were assessed at a single time point at the 12-month follow-up.

- *Perceived impacts of COVID-19* on adolescents and their families were assessed across 12 domains, including: employment status of family members; educational activities of the adolescent; economic conditions (household income and expenditure); food security (access to food and daily necessities); psychological stress related to social distancing; social relationships and support (emotional relationships within the family, visiting relatives and friends, and support from relatives/friends and the community); access to healthcare when ill; and other COVID-19–related disruptions. Each domain-specific impact and the overall perceived impact were rated on a 5-point Likert scale ranging from 1 (“not at all”) to 5 (“extreme”) [9]. Mean scores were calculated for descriptive purposes, with higher scores indicating greater perceived impact.

In addition to domain-specific assessments, participants were asked to rate the *overall perceived impact* of COVID-19 across all domains collectively. Overall impact was summarized using both mean scores and categorical proportions, serving complementary purposes: mean scores reflect the relative severity of perceived disruption, while proportions indicate the share of participants experiencing substantial impact. For regression analyses, responses were dichotomized into “significant impact” (scores 4–5) and “no or insignificant impact” (scores 1–3). This binary measure of overall perceived COVID-19 impact was used as the primary dependent variable to identify factors associated with pandemic vulnerability. This approach is consistent with methods used in similar studies assessing pandemic-related impacts in adolescent populations [13, 14].The threshold was selected because scores of 4–5 explicitly reflected “a lot” or “extreme” disruption, representing a meaningful distinction from no or minimal impact levels.

- *Direct COVID-19 exposure outcomes* were assessed by asking whether the adolescent or any household member had been exposed to or diagnosed with COVID-19 during the study period.

#### Independent variables

Independent variables included characteristics at the adolescent, household, and caregiver levels.

- *Adolescent characteristics* included age, sex, place of residence, clinical stage, ART duration, current ART regimen, and daily ART dosing frequency.
- *Household characteristics* included household size, household structure, and home ownership status.
- *Caregiver characteristics* included age, relationship to the adolescent, educational attainment, employment status, monthly household income, caregiving burden, caregiver fatigue, and reported receipt of family or social support during the pandemic.

### Data analysis

Data were analysed using Stata version 17. Descriptive statistics were used to summarize participant characteristics. Chi-square tests were performed to compare proportions across groups. Candidate variables for multivariable logistic regression were identified from those significant at *p* < 0.05 in univariate analyses, supplemented by a priori clinically relevant factors. Collinearity diagnostics were performed prior to model construction; where strong collinearity was detected (VIF > 5 or |r| > 0.7), the variable with the weaker association with the outcome was excluded. The final model was constructed using the enter method and fit assessed by the Hosmer–Lemeshow test. Results are reported as adjusted odds ratios (aOR) with 95% confidence intervals (95% CI); *p* < 0.05 was considered statistically significant.

### Ethical issues

Written informed consent was obtained from both caregivers and adolescents prior to participation. The study was conducted in accordance with the Declaration of Helsinki and was approved by the Hanoi Medical University Institutional Review Board (Decision No HMUIRBI08/IBB-HDDDNCYSH-DHYHN, 29 April 2020).

## Results

### Participant’s characteristics

A total of 645 adolescents were initially identified, of whom 625 met the inclusion criteria and consented to participate. During follow-up, 38 were referred to local facilities, resulting in a final analytical sample of 587 participants. Among those included, approximately half were male (50.8%). The mean age was 12.8 years (SD = 1.6), with comparable age distributions between sexes. Adolescents aged 13–15 years accounted for 57.4% of the sample. The majority of adolescents (62.0%) resided in the four provinces and cities where the participating hospitals were located, with the largest proportion living in Ho Chi Minh City (33.6%). Most adolescents lived in households with three to four members (54.5%), and 78.5% lived with at least one parent. Additionally, 70.5% resided in family-owned housing.

The mean age at HIV diagnosis was 2.6 years (SD = 2.8), and adolescents had been receiving ART for an average of 9.9 years (SD = 3.0). Nearly all adolescents (99.5%) were classified as WHO clinical stage I, and the majority (89.8%) were receiving first-line ART regimens. Approximately two-thirds of adolescents (63.4%) were taking ART twice daily.

*In terms of primary caregivers*, the mean age was 44.2 years old (SD = 11.3) and approximately half (50.8%) were the adolescents’ mothers. Most primary caregivers had completed secondary school education or lower (64.4%) and reported having stable employment (69.7%). The majority (70.2%) reported a monthly household income below 10 million VND (approximately 425 USD). A substantial proportion of caregivers (75.6%) reported experiencing difficulties in caring for adolescents during the study period. Most did not receive support from family members (68.8%) or external social support (59.8%). More than half (52.0%) of caregivers reported experiencing fatigue while caring for adolescents, with 2.8% indicating frequent fatigue.

### Impacts of COVID-19 on adolescents living with HIV and their families

#### COVID-19 impacts across life domains

**Fig. 1:**
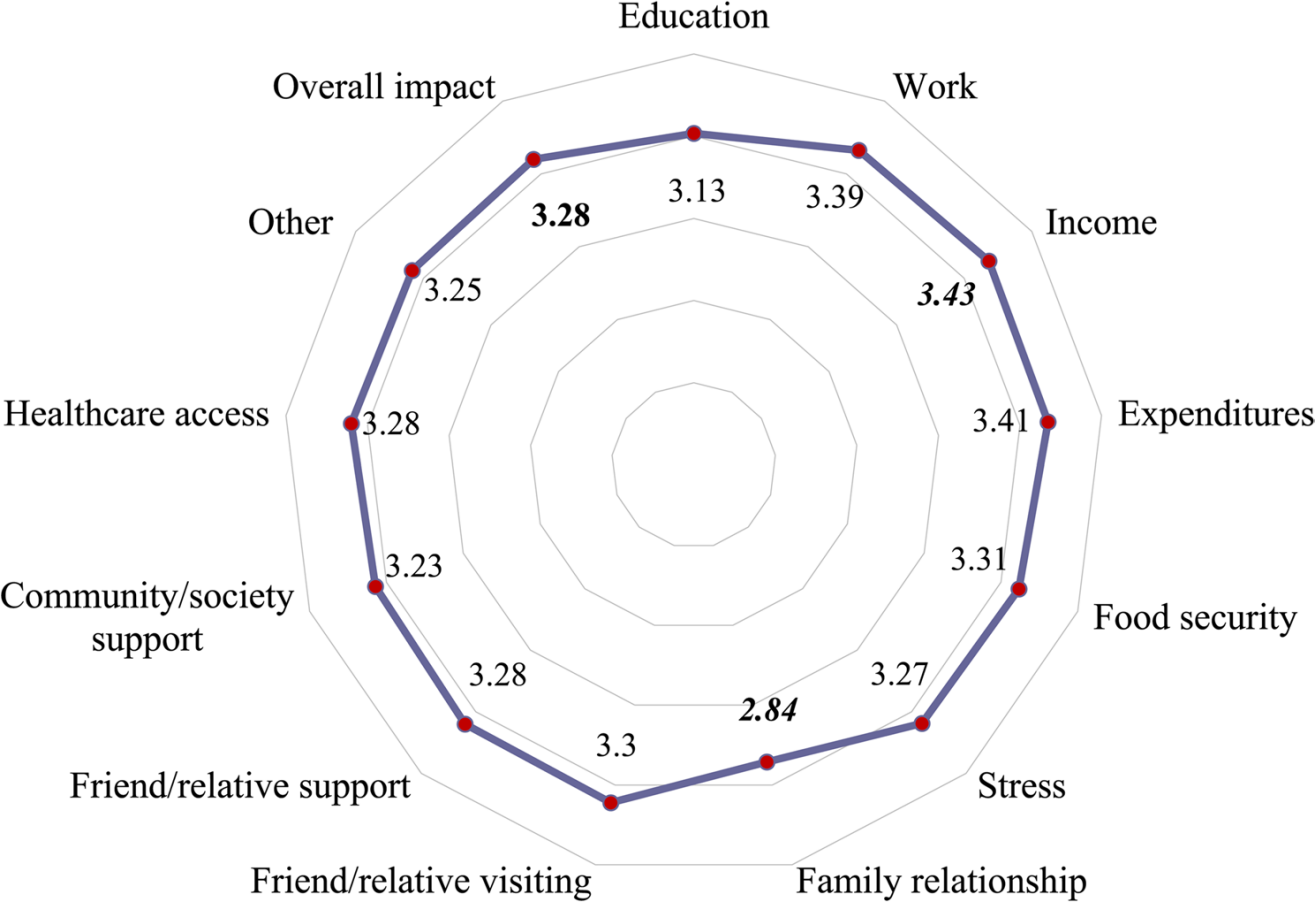
Mean scores of perceived COVID-19 impact across multiple life domains among adolescents living with HIV and their families

The mean score for overall COVID-19 impact was 3.28 out of five. Among the assessed domains, income and expenditure were the most affected, with mean scores of 3.43 and 3.41, respectively. Conversely, family relationships and education were among the least affected domains, with mean scores of 2.84 and 3.13, respectively (Fig. 1).

**Fig. 2:**
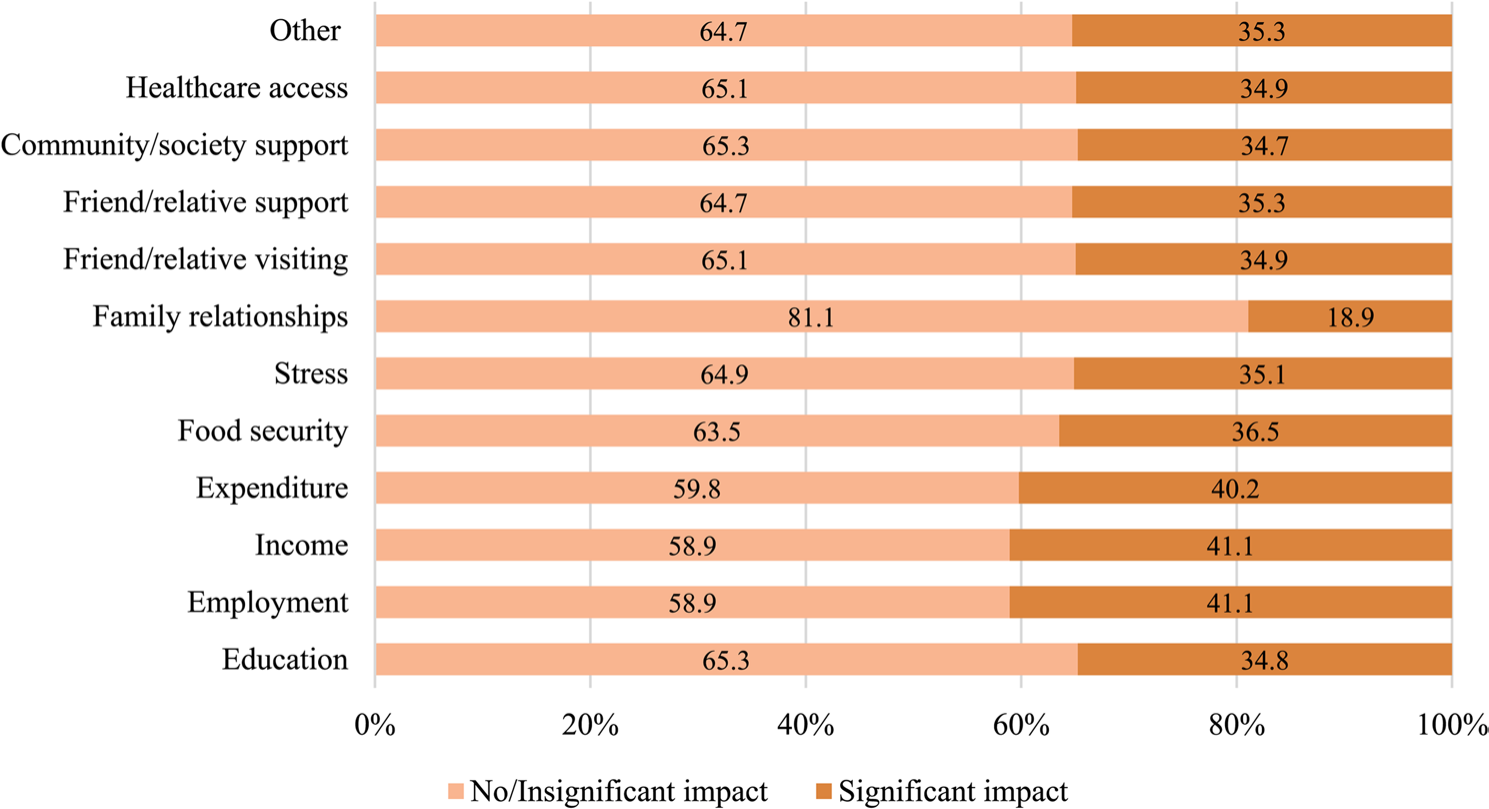
Proportions of adolescents reporting significant COVID-19 impact across different life domains

In Fig. 2, when COVID-19 impacts were dichotomized into significant (score 4–5) versus no or insignificant overall perceived impact (score 1–3), economic domains remained the most affected. The highest proportions of participants reporting a significant overall impact were observed for income and employment (41.06%), and household expenditure (40.2%). In contrast, family relationships and education showed the lowest proportions of significant overall impact, at 18.9% and 34.7%, respectively.

#### The overall perceived impact of COVID-19

**Fig. 3:**
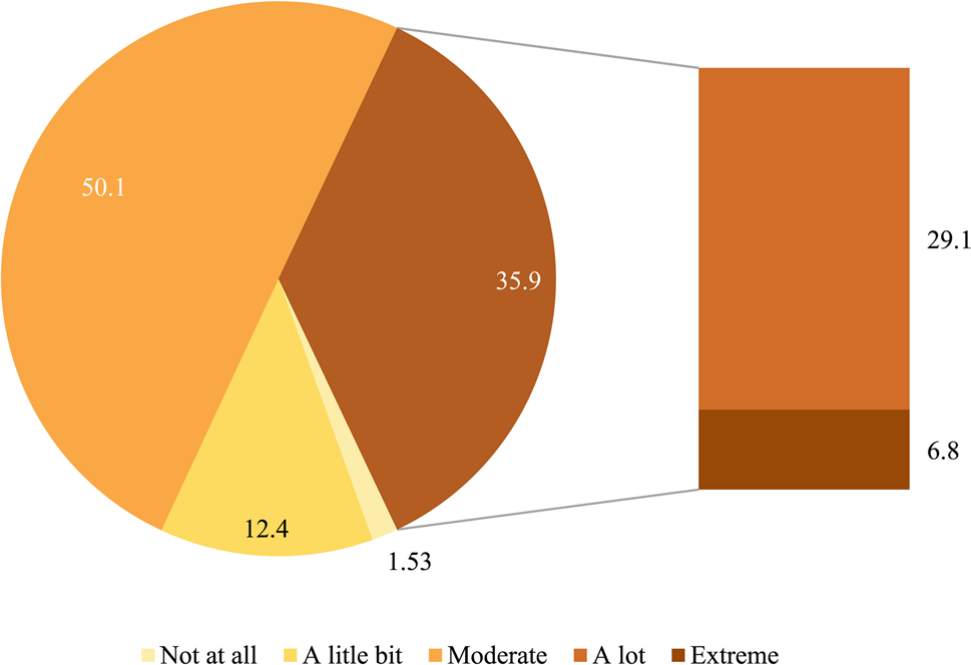
Overall perceived impact of COVID-19 on adolescents living with HIV and their families

Overall, nearly all participants (98.5%) reported experiencing some degree of perceived impact from COVID-19. Half of the participants (50.1%) reported a moderate overall perceived impact, while more than one-third (35.9%) reported a significant overall perceived impact on their lives and those of their families (Fig. 3).

#### COVID-19 direct exposure outcomes

**Fig. 4:**
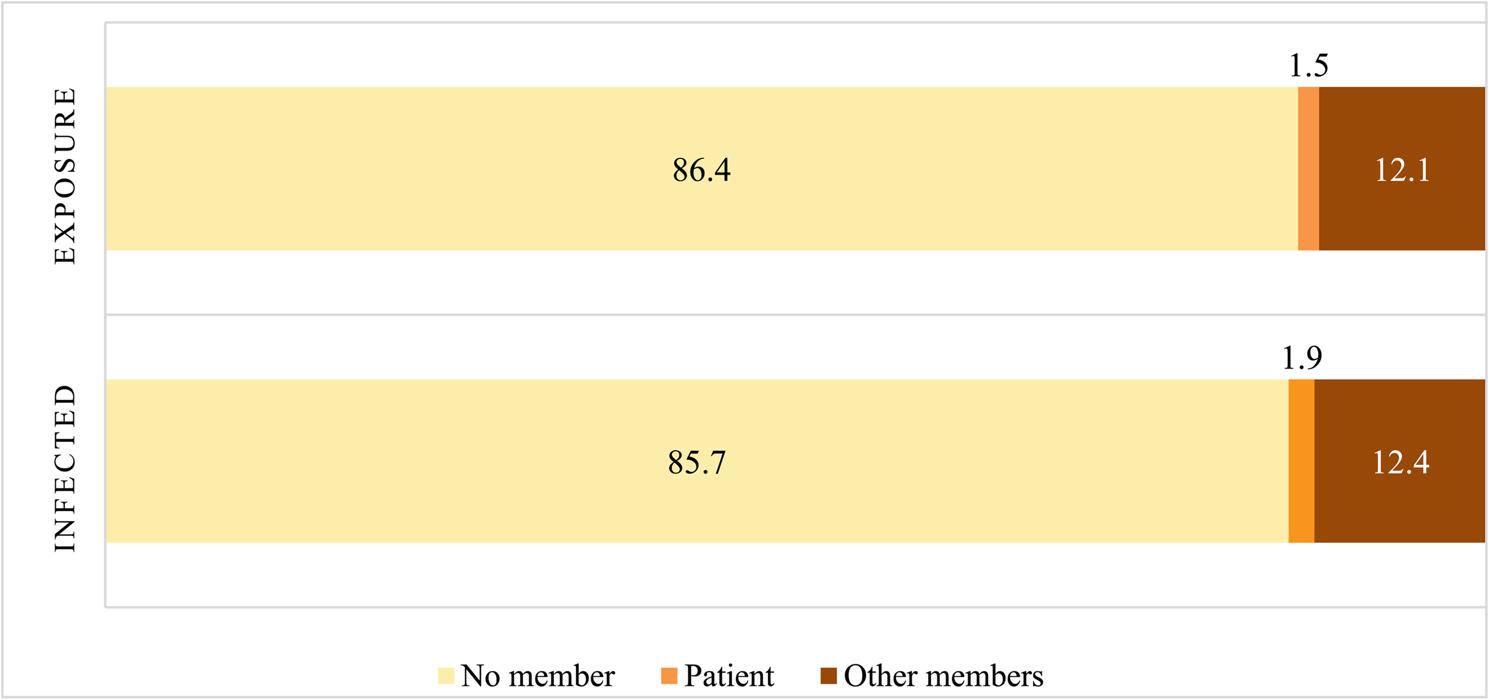
COVID-19 exposure and infection among adolescents living with HIV and their family members

Overall, 13.6% of participants reported COVID-19 exposure and 14.3% reported infection among household members. Among adolescents, exposure and infection were reported by 1.5% and 1.9%, respectively (Fig. 4).

### Factors associated with the overall perceived COVID-19 impact

As shown in Table 1, the proportion of participants reporting a significant overall perceived COVID-19 impact varied across demographic, clinical, and household characteristics. Higher proportions of significant overall impact were observed among adolescents residing in HCMC, those without formal education, those diagnosed with HIV before one year of age, those with an ART duration of less than five years, those taking ART twice daily, those living in households without home ownership, and those living in larger households. Higher proportions were also found among participants whose caregivers’ reported difficulties in caregiving, received family or social support, or experienced caregiver fatigue. In contrast, lower proportions of significant overall perceived impact were observed among caregivers with education above high school level and among households with a monthly income of 5 to < 10 million VND. 

**Table 1:** Overall perceived impact of COVID-19 by demographic, clinical, and family characteristics of participants

Variables			*Significant impact*	*No/insignificant impact*	*p-value*
			*n (%)*	*n (%)*	
*Patient’s factors*	*Province*	Ho Chi Minh City	129 (65.5)	68 (34.5)	**< 0.001**
		Hanoi	4 (4.6)	83 (95.4)	
		Hai Phong	6 (10.5)	51 (89.5)	
		Quang Ninh	4 (17.4)	19 (82.6)	
		Other provinces	68 (30.5)	155 (69.5)	
	*Education*	No	30 (57.7)	22 (42.3)	**< 0.01**
		Yes	181 (33.8)	354 (66.2)	
	*Age at HIV diagnosis*	Under 1 year old	79 (49.1)	82 (50.9)	**< 0.001**
		1–5 years old	95 (27.9)	245 (72.1)	
		Above 5 years old	37 (44.0)	47 (56.0)	
	*ART duration*	Less than 5 years	18 (45.0)	22 (55.0)	**< 0.01**
		5–9 years	50 (27.8)	130 (72.2)	
		10 years or longer	143 (38.9)	224 (61.1)	
	*Current clinical stage*	First stage	210 (36.0)	374 (64.0)	> 0.05
		Second stage	1 (33.3)	2 (66.7)	
	*ART regime*	First level	188 (35.7)	339 (64.3)	> 0.05
		Second level	23 (38.3)	37 (61.7)	
	*Daily ART dosage*	1	40 (18.6)	175 (81.4)	**< 0.001**
		2	173 (46.5)	199 (53.5)	
	*Family size*	1–2 persons	22 (25.0)	66 (75.0)	**< 0.01**
		3–4 persons	109 (34.1)	211 (65.9)	
		5 persons and more	80 (44.7)	99 (55.3)	
	*Family structure*	Patient living with father and/or mother	158 (34.3)	303 (65.7)	> 0.05
		Patient living with grandparent	45 (43.3)	59 (56.3)	
		Patient living with other relatives	8 (36.4)	14 (63.6)	
	*Homeownership status*	Having own house	119 (28.7)	295 (71.3)	**< 0.001**
		Not having own house	92 (53.2)	81 (46.8)	
*Primary caregiver’s factors*	*Relationship with patient*	Mother/Father	146 (35.1)	270 (64.9)	> 0.05
		Others	65 (38.0)	106 (62.0)	
	*Occupation*	Stable job	157 (38.4)	252 (61.6)	> 0.05
		Unstable job	54 (30.3)	124 (69.7)	
	*Education level*	Primary school	76 (46.1)	89 (53.9)	**< 0.01**
		Secondary school	79 (37.1)	134 (62.9)	
		High school	40 (30.8)	90 (69.2)	
		Higher than high school	16 (20.2)	63 (79.8)	
	*Monthly income*	0 - <5 million	95 (35.3)	174 (64.7)	**< 0.05**
		5 - <10 million	42 (29.4)	101 (70.6)	
		≥ 10 million	26 (49.1)	27 (50.9)	
		No answer	50 (41.0)	72 (59.0)	
	*Difficulty in taking care of adolescents*	No	31 (21.8)	111 (78.2)	**< 0.001**
		Yes	182 (41.0)	262 (59.0)	
	*Received any family support*	No	132 (32.7)	272 (67.3)	**< 0.01**
		Yes	81 (44.3)	102 (55.7)	
	*Received any social support*	No	62 (17.7)	289 (82.3)	**< 0.001**
		Yes	151 (64.0)	86 (36.0)	
	*Caregiver’s fatigue status*	Never	72 (29.6)	171 (70.4)	**< 0.001**
		Sometimes	125 (50.2)	124 (49.8)	
		Often	3 (21.4)	11 (78.6)	

Results from the multivariable logistic regression analysis are presented in Table 2. Geographic location was strongly associated with reporting a significant overall perceived COVID-19 impact. Compared with adolescents residing in the reference area, higher odds of significant overall perceived impact were observed among those living in HCMC, Quang Ninh, and provinces outside the hospitals’ catchment areas.

**Table 2:** Multivariable logistic regression analysis of factors associated with significant overall COVID-19 impact

Variables	Significant COVID-19 impact	
	aOR (95%CI)	*p*-value
*The province of the patient’s residence*		
Hai Phong	1.1 (0.3–4.4)	> 0.05
Quang Ninh	5.9 (1.2–28.6)	**< 0.05**
Ho Chi Minh City	18.8 (5.9–60.0)	**< 0.001**
Other provinces	4.9 (1.6–14.9)	**< 0.01**
(Ref: Hanoi)		
* Having education*	0.4 (0.1–1.0)	> 0.05
* Taking 2 daily ART doses*	1.9 (1.1–3.5)	**< 0.05**
*Family size*		
3–4 persons	2.9 (1.3–6.5)	**< 0.01**
5 persons and more	2.7 (1.2–6.1)	**< 0.05**
(Ref: 1–2 persons)		
* Not having own house*	2.0 (1.1–3.3)	**< 0.05**
*Education level of caregiver*		
Secondary school	1.0 (0.6–1.9)	> 0.05
High school	1.1 (0.5–2.2)	> 0.05
Higher than high school	0.6 (0.3–1.5)	> 0.05
(Ref: Primary school)		
* Difficulty in taking care of adolescents*	1.4 (0.7–2.7)	> 0.05
* Caregiver received family support*	1.6 (0.9–2.7)	> 0.05
* Caregiver received social support*	4.6 (2.8–7.7)	**< 0.001**
*Caregiver’s fatigue status*		
Sometimes	1.2 (0.7–2.1)	> 0.05
Often	0.3 (0.1–1.3)	> 0.05
(Ref: Never)		

ART and household related factors were also associated with the overall perceived impact of COVID-19. Adolescents taking ART twice daily had higher odds of reporting a significant overall perceived impact compared with those taking ART once daily. Higher odds were also observed among adolescents living in households without home ownership and those living in households with three or more members.

With respect to caregiver related characteristics, adolescents whose caregivers reported receiving social support had higher odds of experiencing a significant overall COVID-19 impact.

## Discussion

### Impacts of the COVID-19 pandemic on adolescents living with HIV and their families

This study provides empirical evidence of the substantial and multidimensional impacts of the COVID-19 pandemic on adolescents aged 10–15 years living with HIV and their families in Vietnam. Nearly all participants (98.5%) reported some degree of impacts, suggesting that the effects of the pandemic were pervasive even when severe outcomes were not reported. This figure exceeded findings from a national study on the impacts of the COVID-19 pandemic among patients with chronic conditions in Vietnam (91.9%) [15].

More than one-third of participants (35.9%) reported experiencing a significant overall perceived impact, underscoring the heightened vulnerability of this population during public health emergencies. Our finding was higher than that reported in a study conducted among mothers in Bhaktapur, Nepal (21.7%) [9]. Although these populations are not directly comparable, the differences highlight how the combined challenges of adolescence and long-term HIV care may amplify vulnerability to pandemic-related disruptions.

Across specific life domains, economic impacts were among the most prominent. In our study, 41.1% of participants reported significant perceived impacts on household income or employment, a proportion higher than that reported in a study from Kenya, where 35.9% of family members experienced job loss or income reduction [16]. These findings are consistent with evidence from a longitudinal study in New York City, in which nearly all participants reported income declines during the pandemic [17], suggesting that economic instability was a widespread consequence of COVID-19 across diverse settings.

Impacts on food security were reported by 36.5% of participants in the present study, which is slightly lower than the prevalence reported in Kenya, where approximately 40.4% of HIV patients experienced food insecurity during the pandemic [16]. Differences in study settings, measurement tools, and sample characteristics may partly explain this variation. In addition, 35.8% of participants reported a significant impact on family relationships. Qualitative evidence from previous studies has similarly documented increased family strain, including heightened conflict and vulnerability among adolescents living with caregivers during periods of lockdown and school closure [18].

Psychological stress associated with social distancing was significant, with 35.1% of participants reporting significant stress. This finding aligns with the broader literature on the mental health impacts of COVID-19 containment measures. Prolonged periods of isolation and restricted mobility may have been particularly burdensome for adolescents living with HIV, who already face heightened psychosocial vulnerability stemming from stigma, disclosure concerns, and the demands of long-term treatment adherence. Evidence from sub-Saharan Africa and Southeast Asia has documented elevated rates of anxiety and depressive symptoms among adolescents living with HIV during the pandemic, driven in part by disrupted peer networks and school closures [18]. In our study, the relatively lower mean score for family relationships (2.84 out of 5) compared with economic domains may suggest that household cohesion served as a partial buffer against psychological distress; however, this interpretation is limited by the absence of validated mental health instruments. Future studies should incorporate standardized tools, such as the PHQ-A or SCARED, to more rigorously characterize the psychological burden of public health emergencies on this population.

Despite substantial perceived COVID-19 impacts across multiple life domains, the proportion of adolescents infected with COVID-19 in this study remained low (1.9%). This contrasts with earlier studies reporting no confirmed COVID-19 cases among adolescents living with HIV [16, 18]. In contrast, the proportion of adolescents reporting exposure to individuals with COVID-19 (1.5%) was lower than that reported in Enane’s study (2.7%) [16]. These differences may be partly attributable to variations in sample size, study period, and local epidemic dynamics, and further underscore that the primary burden of the pandemic for this population stemmed from indirect social and economic effects rather than direct infection.

### Factors associated with the overall COVID-19 impact

In this study, several demographic, clinical, household, and caregiver-related factors were associated with reporting a significant overall impact of the COVID-19 pandemic. In univariate analyses, higher proportions of significant overall impact were observed among participants residing in HCMC, those with markers of socioeconomic vulnerability, and those whose caregivers reported greater caregiving burden. However, only a subset of these factors remained independently associated with the overall perceived impact after adjustment in multivariable models, highlighting the role of confounding and interrelated social determinants.

Geographic location emerged as a strong correlate of the overall perceived COVID-19 impact. Compared with adolescents living in Hanoi, those residing in HCMC, Quang Ninh, and other provinces outside the hospital catchment areas had significantly higher odds of reporting a significant overall perceived impact. These differences may be attributable to variations in local socioeconomic conditions, COVID-19 transmission and exposure levels, and the intensity and duration of quarantine and lockdown measures implemented across provinces.

The highest odds of reporting a significant overall perceived COVID-19 impact were observed among adolescents living in HCMC (aOR = 18.8; 95% CI, 5.9–60.0). This finding likely reflects the unique pandemic context in HCMC during the study period, when the city experienced the most intense COVID-19 transmission nationwide [19, 20]. Prolonged and stringent lockdown measures, healthcare system strain, and exceptionally high daily case numbers may have amplified disruptions to healthcare access, household livelihoods, and psychosocial well-being among families of adolescents living with HIV. In contrast, although Hanoi and other provinces were also affected by COVID-19, they experienced comparatively lower disease burden and mortality rates during the same period [21]. These contextual differences may partly explain the observed geographic variation in pandemic impacts. Nevertheless, given the cross-sectional design of the study and the wide confidence interval surrounding the estimated odds ratio, residual confounding and unmeasured provincial-level contextual factors cannot be excluded. Accordingly, this association should be interpreted as reflecting a strong contextual relationship rather than a causal effect.

Our study did not identify a statistically significant association between household income and the overall perceived impact of COVID-19 among families of adolescents living with HIV. This finding contrasts with evidence from non-HIV populations, where lower household income has been consistently associated with greater pandemic impacts. For example, a cross-sectional study conducted in Geneva, Switzerland reported that children and adolescents from average-to-poor income households were substantially more likely to experience severe pandemic impacts compared with those from higher-income families (OR = 4.6; 95% CI, 3.2–6.8) [13]. The absence of a similar association in our study may reflect the buffering role of Vietnam’s HIV care and social protection programs, which provide relatively stable access to treatment and essential services regardless of short-term income fluctuations. However, given the cross-sectional design, further longitudinal research is needed to clarify whether household income modifies pandemic-related vulnerability among families affected by paediatric HIV.

Furthermore, in other aspects of child healthcare, such as dental care, Burgette et al. reported that a decrease in income or lower income due to the COVID-19 pandemic was associated with a higher risk of unmet child dental care needs (RR = 1.77; 95% CI, 1.08–2.88) [22]. These findings suggest that the economic consequences of the pandemic disproportionately affected lower-income families, leading to greater unmet healthcare needs. Further studies are needed to examine the relationship between household income and COVID-19 impacts among children living with HIV.

We also found that adolescents taking ART twice daily were significantly more likely to experience a significant overall perceived COVID-19 impact than those taking one daily dose. This association should be interpreted as reflecting treatment complexity and care dependency rather than a direct effect of dosing frequency. In paediatric and adolescent HIV care, regimen complexity is a well-recognized challenge, with frequent dosing and formulation issues identified as barriers to adherence that require sustained caregiver involvement. International guidelines recommend prescribing simplified regimens (e.g., once-daily, low pill burden) when feasible to support optimal adherence in children and adolescents [23]. In this context, more frequent daily ART dosing may serve as a proxy for less simplified regimens, which are likely to require greater caregiver involvement and more consistent healthcare access. This may help explain why greater perceived impacts were reported among adolescents taking ART twice daily in our study. Qualitative studies from resource-limited settings have further documented that complex ART administration routines, including frequent dosing and intricate preparation, contribute to caregiver burden and difficulties in treatment management [24].

During the COVID-19 pandemic, disruptions to healthcare access and increased caregiving burden may have disproportionately affected families managing more complex treatment routines [3, 5]. Accordingly, this finding likely identifies a subgroup with heightened vulnerability rather than implying a causal role of ART dosing frequency. Given the cross-sectional design of the study, this finding should be interpreted as reflecting heightened vulnerability among adolescents receiving more complex ART regimens, rather than implying a causal effect of dosing frequency itself.

In Vietnam, multi-month dispensing (MMD) of ART has been widely implemented since 2019, contributing to treatment continuity during routine care and public health disruptions [25]. Building on this experience, future research may explore whether modest extensions of dispensing intervals or integration with decentralized drug distribution approaches could further enhance treatment continuity for clinically stable adolescents during public health emergencies.

Our study indicates that caregivers who reported receiving social support were more likely to experience a greater overall perceived impact of COVID-19. This association may reflect reverse causality, whereby caregivers facing more severe difficulties actively sought or were offered additional support during the pandemic. Moreover, access to social support during COVID-19 likely depended on the availability and accessibility of resources, which were uneven and frequently disrupted. Similar challenges related to social isolation and disrupted support networks have been documented among people living with HIV in the United States [26]. Taken together, these findings suggest that social support received during crises may reflect heightened underlying need rather than acting as uniformly protective. They underscore the importance of resilient HIV care models and strengthened social protection systems to safeguard adolescents living with HIV during future public health emergencies.

In this study, the likelihood of experiencing a significant overall perceived impact of COVID-19 was nearly three times higher among families with three or more members compared with smaller households. Although the cross-sectional design of the study does not allow causal inference, this association is plausible in the context of the COVID-19 pandemic. Larger household size may be associated with greater financial pressure, increased competition for limited resources, and heightened food insecurity during periods of economic disruption and mobility restrictions.

Several caregiving-related variables were significant in univariate analyses but did not remain so after adjustment, suggesting that their effects may be mediated or confounded by broader household and contextual factors.

### Limitations

Identifying factors associated with overall perceived COVID-19 impact is important for informing preparedness and care planning for future public health emergencies. Nevertheless, several limitations should be acknowledged.

Firstly, although most adolescents living with HIV in Vietnam were concentrated in major paediatric hospitals during the study period, restricting recruitment to large central and provincial hospitals may have introduced selection bias. Although most adolescents living with HIV were managed through outpatient clinics at major hospitals, the small proportion receiving care in decentralized settings or lost to follow-up may have experienced different or more severe disruptions. Therefore, while the risk of selection bias is likely minimal, the generalizability of our findings should be interpreted with caution.

Secondly, the sample size estimation was based on the most contextually relevant evidence available from another low- and middle-income country, as no directly comparable prior data were available for adolescents living with HIV in Vietnam. Accordingly, this prevalence estimate should be considered a pragmatic planning assumption rather than a precise prior parameter.

ThirdlyCOVID-19 impacts were assessed retrospectively using self-reported measures and may be subject to recall bias. Thirdly, mental health–related domains reflected perceived stress rather than clinically validated psychological outcomes, as standardized mental health instruments were not applied.

In addition, dichotomizing the overall perceived impact measure may have reduced variability and should be interpreted as a simplified indicator of perceived disruption. Future studies should consider analysing the full impact score using continuous or ordinal models to allow more nuanced estimation of associated factors, alongside qualitative or mixed-methods studies are also warranted to further explore adolescents’ lived experiences, coping strategies, and psychosocial needs during public health crises.

## Conclusion

The COVID-19 pandemic substantially affected the lives of adolescents living with HIV aged 10–15 years who were receiving ART at large paediatric hospitals in Vietnam, as well as their families. Over one-third of participants reported significant overall disruption, particularly in economic and household-related domains. Higher overall perceived impact was independently associated with several contextual, socioeconomic, and care-related factors, including residence in heavily affected areas, lack of home ownership, larger household size, caregiver need for social support, and twice-daily ART dosing. These findings underscore the need for resilient, adolescent-friendly HIV service delivery, integrated family and psychosocial support, and strengthened social protection and emergency preparedness strategies to better safeguard vulnerable adolescents during future public health crises.

## Supplementary Information

Supplementary Material 1.

## Data Availability

The data presented in this study are available on request from the corresponding author. The data are not publicly available due to ethical application and approval.

## Abbreviations

ART: Antiretroviral therapy
ARV: Antiretroviral
COVID-19: Coronavirus disease 2019
HCMC: Ho Chi Minh City
HIV: Human immunodeficiency virus
aOR: Adjusted odds ratio
RR: Relative risk
SARS-CoV-2: Severe Acute Respiratory Syndrome Coronavirus 2
SD: Standard deviation
WHO: World Health Organization
