# Supplementary material for: Impacts of the COVID-19 pandemic on the lives of adolescents living with HIV aged 10–15 years and their families in Vietnam
Source: BMC Public Health. 2026 May 26;26:1710. doi: 10.1186/s12889-026-27867-3 (PMC13202863; doi:10.1186/s12889-026-27867-3)
Supplement: Supplementary file 1 — Supplementary Material 1. [file 12889_2026_27867_MOESM1_ESM.docx]

*HIVCHI – HANOI MEDICAL UNIVERSITY*

**Trial Protocol**

**A randomized controlled trial to assess the effect of group peer support to children and adolescents with HIV in relation to adherence, virological treatment failure, physical development and quality of life.**


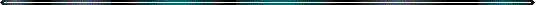


**Principal Investigator (PI):**

Mattias Larsson, Assoc Prof. MD, Nguyen Thi Kim Chuc, Assoc Prof.

**Co-Investigators:**

Linus Olson, PhD, M. Med. Tech, Tran Khanh Toan, Assoc Prof.

**Study statistician:**

**Study Sponsor:**

The Swedish Research Council (Vetenskapsrådet) and European Union, Marie Curie

**Study Sites:**

National Pediatric Hospital in Hanoi

Children’s Hospital number 1 in Ho Chi Minh city

Children’s Hospital number 2 in Ho Chi Minh city

**Protocol Précis**

Globally the estimated number of children living with HIV is 3 million. In Vietnam there are about 7100 children diagnosed with HIV, and 5000 are on treatment. Generally children with HIV are diagnosed late. The estimated average age at HIV diagnosis in the National Pediatric Hospitals is 3 years, and by this time the children are often severely immune-compromised. Children with HIV on ART have about twice the rate of virological treatment failure compared to adults. A large meta-analysis of pooled data from HIV-infected children after 12 months of ART showed that virological treatment failure rate is 30%; among those 13,7% had immunological failure. The consequences of treatment failure is greater in children as antiretroviral options for children are much more limited. The impact of this is further compounded for HIV-infected children in resource-poor settings where access to viral load monitoring is scare, and virological treatment failure is detected late, often long after they have experienced immunological and/or clinical failure. Prolonged treatment of non-suppressive therapy leads to development of accumulated drug resistance mutations and replication of multi-drug resistance HIV species, which further limit future therapeutic choices for children in resource poor settings. Many children live in difficult family situations with drug addiction and/or single mothers who are themselves infected with HIV. The lack of a structured support system of caretakers is probably the single most important factor driving poor treatment adherence in children. Several cross-sectional studies have shown a clear relationship between poor adherence and resistance development. There is great need to develop low cost and sustainable interventions that can be implemented in developing countries to improve adherence and long-term treatment outcome in children on ART.

**Synopsis**

**Title:** A randomized controlled trial to assess the impact of peer support to children and adolescents with HIV receiving antiretroviral therapy (ART) with virological treatment failure as primary endpoint

**Primary Objectives:** To study the impact of peer support to care takers of children with HIV receiving ART vs conventional care on rates and time to virological treatment failure and /or death and/or AIDS-associated events (clinical treatment failure) up to 24 month using a randomized controlled trial (RCT) design comparing enhanced treatment support (ETS) and conventional treatment (CT).

**Secondary Objectives:**

1. To compare the rate of and time to virological treatment failure (primary endpoint) immunological failure (secondary endpoint) /death/AIDS-associated events (tertiary endpoint) between the intervention and control group
2. To compare the rate of ART adherence between the intervention and control group
3. To compare the rate of acquiring OI’s, drug adverse effect, physical development, drug resistance between the intervention and control group.
4. To asses quality of life in intervention and control group before and after intervention
5. To assess cost effectiveness of peer support comparing to conventional care

**Study Designs:**

Randomized controlled trial, multi-center study

Randomization will be 1:1 and stratified by center and ART status at enrolment stratified according to naïve vs. on ART treatment.

**Study Locations:**

- National Pediatric Hospital in Hanoi

- Children’s Hospital 1 in Ho Chi Minh City

- Children’s Hospital 2 in Ho Chi Minh City

**Study Regions:**

- Hanoi and Ho Chi Minh city

**Study Size:**  Planned enrollment of a total of 540 HIV positive children 10-16 years of age, on ART and randomize to either Enhanced Treatment Support (ETS) or Conventional Treatment (CT).

**Study Duration:** Study enrollment at the second quarter of 2019 and the study will be completed when all patients have completed follow up during 24 months.

**Study Population:**

**Inclusion Criteria:**

- Continuously followed up by Out Patient Clinic (OPC).
- Age from 10 to 16 years of age (based on date of birth)

**Exclusion Criteria:**

(any of the following):

- Being referred from other sites for temporarily follow-up at site study.
- Institutionalized patients (e.g. orphanage)

**Sample size:**

WHO sample formula for the intervention study, comparing to 2 proportions

$$n=\frac{\left(Z_{1-\alpha/2}\sqrt{2pq}+Z_{1-\beta}\sqrt{p_{1}q_{1}+p_{2}q_{2})}\right)^{2}}{\left(p_{1}\_p_{2}\right)^{2}}$$

We assume the possibility of treatment failure of 15% in the control arm and 10% in the intervention arm after 2 years of follow-up. With 80% power and a test at the two-sided 5% significance level, 10% losses of follow-up, approximately 540 patients are required **(260 per group)** into the trial.

**Enrollment:** All patients aged 10-18 years meeting the inclusion criteria are eligible at the study sites will be enrolled.

**Randomization:** The patients are randomized in the intervention or control group the first time of the intervention. E.g. all patients that attend the examination at a specific weekday will be included in intervention group other week day will be the control group. The peer group meetings will then be organized according to the day of examination where each group contain about 15 patients. Patients can/will not know which day is which until they come to avoid bias. Written consent will be done.

## Peer Support Intervention “Enhanced Treatment Support (ETC)”

1. Caretakers of children and adolescents with HIV on ART will be supported to achieve better adherence through peer support groups and phone follow-up, with an option for home visits or to return to support groups later if necessary. The activities will be organized by peer supporters, HIV-infected young adults selected by peers who will undergo a series of training, including medication adherence and counselling skills.

The support will include

1. Peer group support:

- Home visit
- Telephone support
- Focus group discussion

1. More support during medical visit
2. Peer group meetings with about 15 children and adolescents and their supporters arranged according to age groups of children and adolescents 10 – 12 years , 16 years as well as according to location, e.g. one group per age group per district. Each peer group meeting will be arranged by two peer supporters, one that takes care of the caretakers and the other the children. Each peer group meeting should have a generic part in relation to adherence where each caretaker can bring up problems and discuss them with the supporter and other group members. At each meeting some new information will be provided, either from the supporter or by some invited person arranged by the project, according to a training program.
3. The supporters will also have at least one telephone contact with the caretakes per week, if needed more, to assess the condition of the child/adolescent. To those who cannot make a particular support group session phone calls or home visits (if consent is given) are made. Follow up phone calls will be made to those whom the case manager identifies to need more support.
4. The meeting will be arranged in relation to the treatment day, e.g. at ND1 on Mondays and Wednesdays, when the patients are available at the clinic.
5. The peer-supporters will be trained by the research team and given training material to share with their Peer support groups. The training will include: Basic HIV knowledge review, Nutrition and HIV, Opportunistic Infections, Mental health, Antiretroviral Therapy and Adherence, Medication Adherence Support, Role of the peer-supporters, Setting boundaries and working as part of multidisciplinary team, Disclosure, Outreach overview, Confidentiality, Counseling skills, Study forms (CRF) and reports
6. In ***CT*** ***control group*** the treatment will be supported according to the treatment guidelines. Both groups will receive treatment counseling and be followed through regular clinical check-ups, the drugs will be provided in a pre-packed dosage form for easy remembering and counting of the pills.

*** Criteria for Peer-supporters:**

- Being HIV positive on ART, caretakers or social workers

- Being in good health condition

- Having good social skills

- Not having their children in study cohort.

- Living nearby or in the study regions.

*** Criteria for Social work supporter:**

- Having good social skills

- At least 2 years expertise of working with social support for HIV patients

*** Training curriculum for Peer-supporters and social work supporter:**

Session 1: Clinical Trial

Session 2: Basic HIV review
Session 3: Nutrition and HIV
Session 4: Opportunistic Infections
Session 5: Mental health
Session 6: Antiretroviral Therapy and Adherence
Session 7: Medication Adherence Support
Session 8: Role of the peer-supporters and social worker
Session 9: Setting boundaries and working as part of multidisciplinary team
Session 10: Disclosure
Session 11: Outreach overview
Session 12: Confidentiality
Session 13: Counseling skills
Session 14: Electronic study forms (CRF) and reports

Assignments for peer supporters

|  | **EST** | **CT** |
| --- | --- | --- |
| 1. Take part in the OPC management children/adolescent on ART(*) | Yes | Yes |
| 2. Group support meetings | Once per month per group | No |
| 2.2 Adherence assessment and reinforcement | Yes, over the phone | No |
| 2.3 Psychological support | Yes, over the phone | No |
| 2.4 Home visit once per month | Yes, and over the phone | No |
| 2.5 Arrange automatic reminder to give/take medicine | Twice a day depending on treatment regiment | No |
| 2.6 Telephone support to confirm time for home visit (at least 3 days in advance) and ask about the status of the patient. | One phone call weekly. Availability for the patient to call the supporter during working time and if emergency. |  |
| 2.7 Disclosure assessment and support | Yes | Yes, if needed |

*Note: (*) according to national guidelines*

**Indicators for Evaluation:**

The primary endpoint is time to virological treatment failure; the secondary endpoints are time to immunological and clinical treatment failure. In addition adherence will be assessed, risk factors of treatment failure will be evaluated and ART resistance mutations will be assessed. Adverse events including ARV adverse drug reactions, immune reconstruction inflammatory syndrome (IRIS) and OIs development, as well as Death/Mortality will be assessed. Loss of follow-up will be determined after the follow up period for patients who do not attend clinical visits and laboratory monitoring and will be analyzed as death. According to intention to treat patients that are absent one or more follow up visits and then turn up will be included in the analysis. Cost effectiveness will be assessed if there is a significant difference after two years follow up between the intervention and control group using costing analysis.

| No. | Indicator | Method of measurement | Method of assessment |
| --- | --- | --- | --- |
| 1 | Time to Virological treatment failure  (primary endpoint) | Viral load test:  -Baseline at enrollment  -Every 6 months of follow-up period | Cutoff (1): 1000 cps/ml (International)  - If the viral load Is above the cut off in the first assessment after 6 months of ART, it is defined as primary virological treatment failure, if the viral load is first suppressed and then detectable, it is referred as secondary virological treatment failure. |
| 2 | Time to Immunological treatment failure (secondary endpoint) | CD4 count:  - Recent result or baseline at enrollment.  Not part of routine follow up but if indicated due to virologic failure. | *Cutoff : below the level of severe immunodeficiency by age  -Child<11 months: % CD4<25%  -12 month <Child<35 months: %CD4<20%  - 35 months <Child<5 years: %CD4<15%  -Child>5 years: CD4 < 100 cell/mm3  *CD4 count falls to or below cutoff after initial recovery response (1). OR CD4 count falls rapidly below cutoff by age as confirmed by at least 2 consecutive times (2). OR CD4 count falls below more than 50% of the peak level during ARV treatment (3).  Decide %, the time length leading to immunological treatment failure in both group (EST & CT) |
| 3 | Clinical treatment failure (tertiary endpoint) | - Physical exam  - WHO scale in infant development (weight, height, physically active) | - Lack of or decline growth rate in children who initially respond to treatment (1).  - Loss of neurodevelopmental milestone or development of encephalopathy (2).  - Occurrence of new OIs or malignancies or recurrence of bacterial or fungal infections that refractory to treatment (3).  Decide %, the time length leading to clinical treatment failure in both group (EST & CT) |
| 4 | Adherence assessment | - Interview  - Questionnaire  - Pill count  - Pharmacy record | * Cut-off: (Good > 95%, Poor <95%)  - Good adherence: > 95% doses taken on time  - Poor adherence: < 95% doses taken OR > 95% doses taken but not on time.  * Other factors related:  - On time at every check-up visit  - Same caretaker at every check-up visit  - The current belief of caretaker to ART  - The current collaboration of child to ART  Decide % adherence in both group (EST & CT) |
| 5 | Risk factors of treatment failure | - Questionnaire  - Counseling | - Decide the pattern of risk factors of both groups and compare. |
| 6 | ART mutation assessment | - Genotyping (sequencing or real-time PCR for specific allele) | * ART mutation assessment for patients that have detectable viral load above 1000 copies/ml  * Baseline resistance will be assessed in a subsample of the cohort.  * Compare to Stanford HIV mutations database regarding to the regimens that patients were treated.  Decide pattern of mutation, % in both group (EST & CT) |
| 7 | ARV adverse effects | - Physical exam  - Lab test (enzyme) | Decide % of children having ARV adverse effects from both group (EST and CT) |
| 8 | IRIS | - Physical exam | Decide % of children having IRIS from both group (EST and CT) |
| 9 | OIs development | - Physical exam | Decide % of children having OIs development from both group (EST and CT) |
| 10 | Death/Mortality | - Regardless death causes | Decide % of children death from both group (EST and CT) |
| 11 | Development of AIDS events | - Physical exam | Decide % of children with development of AIDS events from both group (EST and CT) |
| 12 | Loss of follow-up  = Death (in analysis) | - Not able to manage within 6 months. | Assume as death/mortality in analysis.  These patients will be drawn out of study, If they come back to OPC, they will receive support as conventional procedure. |
| 13 | Physical development | Develop according to normal development |  |
| 14 | Quality of Life | - Assessment by caretakers and patients. | Using the PedQoL questionnaire adapted for different ages. |
| 15 | Cost effectiveness | - Collecting monetary data of expenses | - Cost: compare between EST to CT  - Effectiveness: % of patients with virological treatment failure |

## Evaluation

- Primary Endpoint: time to virological treatment failure and /or death and/or AIDS-associated events (clinical treatment failure)

## Statistical Considerations

- The statistical analysis mainly consists in the comparison between the two groups ETS and CT with respect to the defined primary and secondary endpoints in relation to the semi-quantitative assessment of adherence (by questionnaire, pill counts, and pharmacy records).
- Standard statistical methods will be used. Hazard/Survival analysis will be conducted including death/loss of follow up as “failure” in the analysis. Intention to treat will be used for analysis.
- Regression models with outcome as the dependent variable and a group indicator together with patient characteristics as independent variables will be the main approach.
- For binary outcomes, logistic regression will be used.
- Attention has to be paid to the particular, often skewed, distributions of variables like viral load. Transformations or the use of non-parametric approaches are likely to be necessary if such measures are not dichotomized and used in linear regression.
- The correlation between drug resistance and adherence will be statistically analysed in order to study whether the basic mechanisms of selective drug pressure result in a concave or bell-shaped resistance – adherence relationship (Friedland and Williams, 1999), for these drugs during ETS or CT in a low-income setting.
- The odds ratios of having or not baseline drug resistance mutations as detected by population and deep (single allele PCR) sequencing on drug resistance development and clinical outcomes during treatment independent of treatment adherence will be evaluated.
- Stratified analysis will be conducted comparing the outcomes among the treatment groups.

## Interval Assessments

### Interval history and physical exam

The Procedure of following up both groups EST (every 6 months/24-36 months of follow-u) and CT (as OPC routine procedure according to national guidelines)

- Vital signs and weight
- Physical examination
- Physics development and awareness
- Signs and symptoms of OIs

etc.

### Adherence Intervention and following-up

- Group CT: Adherence assessment and Support as in conventional procedure every 6 months
- Group EST:

- Adherence assessment and Support as in conventional procedure every 6 months

- Additional adherence assessment and Use Peer-Support as in study procedure (peer-support weekly)

### Interval laboratory test

Patients of 2 groups will be followed for 24 months and monitored every 6 months for viral load. Other laboratory tests schedule as in National guidelines.

### Archived plasma for further analysis

Left plasma from routine clinical or research laboratory tests will be stored at -70ºC for further analysis with higher techniques by sending out of Vietnam etc. shared outside the clinical and research team.

# Data Management

Briefly, source documents will be generated during the study by the site study staffs at participating institutions including all recordings of observations or notations of clinical activities, and all reports and records necessary for the evaluation and reconstruction of the clinical trial. Source documents include, but are not limited to, the subject’s medical records, laboratory reports, x-rays, radiologist’s reports, subject’s diaries, biopsy reports, ultrasound photographs, progress notes, pharmacy records, and any other similar reports or records of procedures performed during the subject’s participation in the study.

Access to applicable source documents will need to be made available for study purposes. The site investigators are responsible for maintaining any source documentation related to the study. Source documentation should support the data collected on the online CRF**,** and must be signed and dated by the person recording and/or reviewing the data. Source documentation must be available for review or audit by the sponsor or designee and any applicable national authorities.

Online Case Report Forms (CRFs) will be used as a data collection tool. The study team will transfer the information from the source documents onto the Online CRFs. CRFs may be used as source documents if they are the primary data collection tool for specified data as documented in written standard operating procedures. The site Investigators are responsible for maintaining accurate, complete and up-to-date records and for tracking receipt of CRFs for each participant. These forms are to be completed on an ongoing basis during the course of the study by authorized individuals.

# Monitoring

## Study Monitoring

The trial will be conducted in compliance with this protocol, International Conference on Harmonization (ICH) Guidelines for Good Clinical Practice (GCP) and any applicable regulatory requirement(s).

As per ICH-GCP 5.18 clinical protocols are required to be adequately monitored by the study sponsor. Monitors, research staff and possibly research nurses at the study sites, will visit the clinical research site to monitor all aspects of the study in accordance with the appropriate regulations and the approved protocol. The objectives of a monitoring visit will be: 1) to verify the existence of signed informed consent documents for each monitored subject; 2) to verify the prompt and accurate recording of all monitored data points, and prompt reporting of all unexpected SAEs; 3) to compare abstracted information with individual subjects’ records and source documents (subjects’ charts, laboratory analyses and test results, physicians’ progress notes, nurses’ notes, and any other relevant original subject information); and 4) to ensure protection of study subjects, investigators’ compliance with the protocol, and completeness and accuracy of study records. The monitors also will inspect the clinical site regulatory files to ensure that regulatory requirements (Office for Human Research Protections-OHRP) and applicable guidelines (ICH-GCP) are being followed. During the monitoring visits, the investigator (and/or designee) and other study personnel will be available to discuss the study progress and monitoring visit.

## Data and Safety Monitoring Plan

An independent data and safety monitoring board (DSMB) will oversee the trial. The DSMB will perform interim analyses annually within study period and will be provided with summary tables of selected adverse events and an analysis of mortality.

Based on these data, the committee has to make one of the following recommendations:

- - - Continue the trial without modification
    - Continue the trial with modification
    - Discontinue the trial due to safety concerns

The DSMB may also suggest discontinuation if the trial results indicate “beyond reasonable doubt” that one of the allocated strategies is better than the other in primary outcome.

As the dissemination of preliminary summary data could influence the further conduct of the trial and introduce bias, access to interim data and results will be confidential and strictly limited to the involved statistician and the monitoring board and results (except for the recommendation) will not be communicated to the outside and/or clinical investigators involved in the trial.

# Human Subject Protections

## IRB Approval

This protocol, patient information sheet, informed consent document, relevant supporting information will be submitted to the designated IRB/EC and should be approved before the study is initiated.

Any amendments must also be approved by the designated IRB/EC prior to implementing changes in the study.

## Compliance With Good Clinical Practice

This study will be conducted in compliance with the conditions stipulated by the Ethical Committee of the Viet Nam Ministry of Health as well as International Conference on Harmonisation /Good Clinical Practice standards (ICH/GCP) Guidelines. In addition, all local regulatory requirements will be adhered to, in particular those which afford greater protection to the safety of the trial participants.

## Informed Consent

The informed consent for this study will be translated into Vietnamese and must be signed by the study participant or legal representative before participation in the study, including any screening procedures. A copy of the signed consent must be provided to the study participant. Signed consents must remain in each study participants study file, and be available for verification by study monitors at any time.

In the case of illiterate subjects, the consent will be read in Vietnamese to the subjects.

If the subject is too ill to consent, the next of kin may consent for the subject. Once the subject is able, the subject will be consented for continuation in the study.

## Rationale for Research Subject Selection

### Inclusion of children from 10 - 16 years of age

- The study will only include children from 10 - 18 years of age with 24 months of follow up, during the study they will reach a maximum of 18 years of age and still be under Pediatric OPC management.

### Justification of Exclusion criteria

- The exclusion criteria “Being referred from other sites for temporarily follow-up at site study” is primarily to avoid loss of follow up since they are not under OPC management.
- The exclusion criteria “Institutionalized patients (e.g. orphanage) “ as they have institutional provision of treatment on regular times

## Record Retention

The investigator is responsible for retaining all essential documents listed in the ICH Good Clinical Practice Guideline. All essential documentation for all study subjects are to be maintained by the investigators in a secure storage facility for a minimum of three years. These records are also to be maintained in compliance with IRB/EC retention requirements, whichever is longest. All stored records are to be kept confidential. It is the investigator’s responsibility to retain copies of source documents

## Storage of Samples (if applicable)

Approximately 2 ml of plasma from each visit will be stored in the hospital freezer at -70ºC only for further analysis. Samples and data will be stored using codes (not subjects’ names) assigned by the investigators. Only investigators will have access to the samples and data. At the end of the study, samples will continue to be stored for at least five years in the hospital freezer at -70ºC.

Subjects may decide at any point not to have their samples stored. In this case, the principal investigator will destroy all known remaining samples and report what was done to the subject.

## Anonymity and Confidentiality

The information obtained during the conduct of this clinical study is confidential. The results of the research study may be published, but patient names or identities will not be revealed. Records will remain confidential. To maintain confidentiality, the principal investigators at each site will keep records in locked cabinets and the results of tests will be coded to prevent association with the subject’s names.

**Data ownership and dissemination policy**

The data collected in each study site is the property of that study site and any use for e.g. publications need the study sites approval. Hanoi Medical University and Karolinska Institutet will compile the whole data sets and analyze. Publications based on this data should have at least one and maximum two co-author from each study site that has contributed according to the Vancouver rules. All other contributing people will be mentioned under study group or acknowledgements.

Reference

1. Jane M.Simon – Review-Antiretroviral Adherence Intervention, Volume 11 Issue 6 Nov/Dec 2003

2. Chirag A.Shah – Adherence to HAART in Pediatric Patients infected with HIV: Issues and Interventions, Indian Journal of Pediatrics Volume 74 – Jan 2007

3. Guidelines for the Use of Antiretroviral Agents in Pediatric Infection.

4. National Guidelines for HIV treatment

5. Predictors of adherence to antiretroviral therapy among HIV-infected persons: a prospective study in Southwest Ethiopia

6. Kamya MR. Mayanja-Kizza H, Kambugu A, et al. academic alliance for AIDS Care and Prevention in Afirca. Predictors of long-term viral failure among Ugandan children and adults treated with antiretroviral therapy. J. Acquir Immune Defic Syndr 2007; 46: 187-93.

7. *Sungkanuparph*, S., Manosuthi, W., Kiertiburanakul, S., et al., *2007*, “Options for a secondline antiretroviral regimen for *HIV* type 1-infected patients whose initial regimen of a fixeddose combination of stavudine, lamivudine, and nevirapine fails. Clin Infect Dis 2007; 44:447-452.

8. Rachel C.Vree – A Systematic Review of Pediatric Adherence to Antiretroviral Therapy in Low-and Middle-Income Countries, The Pediatric Infectious Disease Journal, Volume 27, Number 8, August 2008.

9. Rache Jean-Baptiste, Factors associated with adherence to antiretroviral therapy in Rwanda: a multi-site study, USAID study 2008.

10. Cheikh Ibrahima Niang and Paul Quarles Van Ufford,- The socio-economic impact of HIV/AIDS on Children in a low prevalence context: the case of Senegal, UNICEF-IRC, Florence, June 2002.

Collett D, Modelling Survival Data in Medical Research. Chapman and Hall/CRC; 2 edition (2003). [Chapter 10]

**Appendix 1A: Study Flow Diagram**

**Consent**

**Inclusion Criteria**

**Exclusion Criteria**

**Baseline assessment**

**Treatment Randomization (1:1)**

**EST**

**CT**

**Peer Support Intervention**

**Follow-up 24 months**

**Analysis**

**Appendix 1 B: Trial Flow Chart for both groups (EST and CT)**

|  |  |  | **24 months follow-up**  **Every 6 months = 6 interval assessment** | | | |
| --- | --- | --- | --- | --- | --- | --- |
| **Event** | **SCR** | **Baseline or enrollment** | **1^st^** | **2^nd^** | **3^rd^** | **4^th^** |
| Informed Consent | x |  |  |  |  |  |
| Inclusion/Exclusion Criteria | x |  |  |  |  |  |
| Medical History | x |  |  |  |  |  |
| Clinical Assessments (vital signs, weight, physical exam) |  | x | x | x | x | x |
| Medication Adherence Assessment (if under ART already) |  | x | x | x | x | x |
| Adverse Event Assessment |  | x | x | x | x | x |
| CBC |  | x | x | x | x | x |
| Liver Enzymes Tests |  | x | x | x | x | x |
| AFB sputum (if available) |  | x | If (indicated) | | | |
| CD4 (if available) |  | x |  |  |  |  |
| Viral load |  | x | x | x | x | x |
| Genotype resistance  (0.5 ml plasma) |  | x | If (indicated) | | | |
| -80 oC Stored plasma  (0.5 ml plasma) |  | x | X | X | x | x |

Volume of Blood sample: 3-4 ml/time, assuming having 1.5 ml of plasma enough for VL, genotypic and storage

**Questionnaires**

**A. Patient’s demographic characteristics, clinical features and ARV treatment**

The following questions are related to the personal information of the child. Please select the appropriate answer or provide the requested information:

**A1**. Patient’s gender: *☐ 1. Male ☐ 2. Female*

**A2**. Date of birth: _ _/_ _/_ _ _ _ (day – month – year)

**A3.** Province of residence

*☐ 1. Hanoi ☐ 2. Hai Phong ☐ 3. Quang Ninh*

*☐ 4. Ho Chi Minh City ☐ 5. Other provinces*

**A4**. Type of education

*☐ 1. No education*

*☐ 2. Home education*

*☐ 3. Attending school*

*_________________________________*

**B. Family’s demographic and socioeconomic characteristics**

The following questions pertain to the information about the child's family. Please select the appropriate answer or provide the requested information:

**B1.** Number of members in the child's family

*☐ 1. 1 – 2 persons ☐ 2. 3 – 4 persons*

*☐ 3. 5 persons or more*

**B2.** Does the current family have your own house?

*☐ 1. Yes ☐ 2. No*

**B3.** Number of family members living with HIV, excluding children: _____ person

______________________________________________________________

**C. Caregiver’s demographic and socioeconomic characteristics**

The following questions relate to the information about the caregiver. Please select the appropriate answer or provide the corresponding response:

**C1.** Date of birth of the caregiver: _ _/_ _/_ _ _ _( day - month - year))

**C2**. Gender of the caregiver:

*☐ 1. Male ☐ 2. Female*

**C3.** Relationship of the caregiver to the child:

*☐ 1. Mother ☐ 2. Father ☐ 3. Grandmother*

*☐ 4. Grandfather ☐ 5. Others*

**C4.** Education level

*☐ 1. Illiteracy ☐ 2. Basic literacy (able to read and write)*

*☐ 3. Primary school ☐ 4. Secondary school*

*☐ 5. High school ☐ 6. Vocational, college, university of higher*

*☐ 7. Don't know*

**C5.** Primary occupation of the caregiver

*☐ 1. Unemployed ☐ 2. Seasonal work*

*☐ 3. Agriculture ☐ 4. Manual labor*

*☐ 5. Office worker ☐ 6. Business*

*☐ 7. Homemaker ☐ 8. Student*

*☐ 9. Other, specify:______________________________*

**C6.** Average monthly income of the caregiver

*☐ 1. Below 5 million VND ☐ 2. 5 – <10 million VND*

*☐ 3. 10 million VND and above ☐ 4. Not known/No answer*

*______________________________________________________________*

**D. Caregivers’ challenges and supports**

The following questions relate to the difficulties faced by caregivers in caring for children with HIV undergoing ARV treatment, and the support received from family and society. Please select the appropriate answer or provide the corresponding response:

**D1.** Have you ever faced difficulties in caring for patient undergoing ARV treatment?

*☐ 1. Yes ☐ 2. No*

**D2.** Have you received any support from family or friends in caring for patient?

*☐ 1. Yes ☐ 2. No*

**D3.** Have you received any support from the community or society in caring for patient?

*☐ 1. Yes ☐ 2. No*

**D4.** Have you ever felt tired, overwhelmed, or desperate when caring for patient?

*☐ 1. Not at all ☐ 2. Sometimes*

*☐ 3. Always ☐ 4. Don't know/No answer*

*______________________________________________________________*

**E. The impacts of COVID-19 pandemic**

In the past 12 months, how has the COVID-19 pandemic affected on your daily life from the following issues?

|  | **Not at all** | **A little bit** | **Moderate** | **A lot** | **Extreme** |
| --- | --- | --- | --- | --- | --- |
| **E1.** Children's education | 1 | 2 | 3 | 4 | 5 |
| **E2.** Employment of family members | 1 | 2 | 3 | 4 | 5 |
| **E3.** Household income | 1 | 2 | 3 | 4 | 5 |
| **E4.** Household expenditure | 1 | 2 | 3 | 4 | 5 |
| **E5.** Food and daily necessities | 1 | 2 | 3 | 4 | 5 |
| **E6.** Stress due to social distancing | 1 | 2 | 3 | 4 | 5 |
| **E7.** Relationships and emotional connections among family members | 1 | 2 | 3 | 4 | 5 |
| **E8.** Visiting friends and relatives | 1 | 2 | 3 | 4 | 5 |
| **E9.** Support from family and friends | 1 | 2 | 3 | 4 | 5 |
| **E10.** Support from community/society | 1 | 2 | 3 | 4 | 5 |
| **E11.** Medical check-ups and treatment | 1 | 2 | 3 | 4 | 5 |
| **E12.** Other, please specify: _________ | 1 | 2 | 3 | 4 | 5 |
| **E13.** Overall impact on all aspects | 1 | 2 | 3 | 4 | 5 |

**E14.** In the past 12 months, has anyone in the child's family been diagnosed with Covid-19?

*☐ 1. No ☐ 2. Yes, the child*

*☐ 3. Yes, another family member*

**E15.** In the past 12 months, has anyone in the child's family had contact with a person infected with Covid-19

*☐ 1.* *No ☐ 2. Yes, the child*

*☐ 3. Yes, another family member*

***Thank you and the interview is concluded./.***
